# Influence of Isothermal ω Transitional Phase-Assisted Phase Transition From β to α on Room-Temperature Mechanical Performance of a Meta-Stable β Titanium Alloy Ti−10Mo−6Zr−4Sn−3Nb (Ti-B12) for Medical Application

**DOI:** 10.3389/fbioe.2020.626665

**Published:** 2021-01-20

**Authors:** Jun Cheng, Jinshan Li, Sen Yu, Zhaoxin Du, Xiaoyong Zhang, Wen Zhang, Jinyang Gai, Hongchuan Wang, Hongjie Song, Zhentao Yu

**Affiliations:** ^1^State Key Laboratory of Solidification Processing, Northwestern Polytechnical University, Xi'an, China; ^2^Shaanxi Key Laboratory of Biomedical Metal Materials, Northwest Institute for Nonferrous Metal Research, Xi'an, China; ^3^School of Materials Science and Engineering, Inner Mongolia University of Technology, Hohhot, China; ^4^State Key Laboratory of Powder Metallurgy, Central South University, Changsha, China; ^5^School of Material Science and Engineering, Northeastern University, Shenyang, China; ^6^Institute of Advanced Wear and Corrosion Resistant and Functional Materials, Jinan University, Guangzhou, China

**Keywords:** phase transition, aging treatment, α precipitated phases, tensile properties, biomedical Ti alloy

## Abstract

The microstructural evolution and tensile performance of a meta-stable β-type biomedical Ti−10Mo−6Zr−4Sn−3Nb (Ti-B12) alloy subjected to one-stage aging (OSA) and two-stage aging (TSA) are investigated in this work. The OSA treatment is performed at 510°C for 8 h. The TSA treatments are composed of low-temperature aging and high-temperature aging. In the first step, low-temperature aging is conducted at 325°C for 2 h. In the second step, the aging temperature is the same as that in the OSA. The result of the microstructure evolution shows that the precipitated secondary phase after aging is mainly influenced by the process of phase transition. There is a marked difference in the microstructure of the Ti-B12 alloy subjected to the OSA and TSA treatments. The needle-shaped α phases are precipitated in the parent β phase after the OSA treatment. Conversely, the short shuttle-like α phases precipitated after the TSA treatment are formed in the β matrix with the aid of the role of the isothermal ω transitional phase-assisted phase transition. The electron backscattered diffraction results indicate that the crystallographic orientation relationship of the α phases precipitated during the TSA treatment is basically analogous to those in the OSA treatment. The relatively higher tensile strength of 1,275 MPa is achieved by strengthening the effect of the short shuttle-like α precipitation with a size of 0.123 μm in length during the TSA treatment, associating with a suitable elongation of 12% at room temperature simultaneously. The fracture surfaces of the samples after the OSA and TSA treatments indicate that preventing the coarsening of the α layers in the grain boundaries is favorable for the enhancement of strength of Ti-B12 at room temperature. MTT test was carried out to evaluate the acute cytotoxicity and biocompatibility of the implanted material using L929 cells. The relative proliferation rates of cytotoxicity levels 0, 1, 2, 3, and 4 are ≥100, 80–99, 50–79, 30–49, and 0–29%, respectively. The cytotoxicity of the Ti-B12 alloy is slightly better than that of the Ti−6Al−4V alloy, which can meet the requirements of medical materials for biomedical materials.

## Introduction

In comparison to α and (α + β) Ti alloys, meta-stable β-type Ti alloys possess outstanding comprehensive mechanical performance (Weiss and Semiatin, 1998; Banerjee and Williams, 2013; Zhu et al., 2016; Zhang et al., 2017; Kaur and Singh, 2019). They have been successfully applied in the aerospace, biomedical, marine, and weapon industries for several decades, owing to their superior biocompatibility, low modulus, corrosion resistance, specific strength, and processability (Cui et al., 2011; Niinomi et al., 2012; Guo et al., 2013; Zhang and Chen, 2019). The higher tensile strength at room temperature could be achieved through the use of aging with a lower temperature due to the finer-scale secondary phases precipitated in the parent β phase for β-type titanium alloys. Moreover, a microstructural configuration with finer, dispersive, and more uniformly distributed α precipitates at the prior β grain boundaries is considered to optimize and enhance the mechanical performance and microstructure stability for the vast majority of β-type titanium alloys (Tang et al., 2000; Qazi et al., 2005; Chen et al., 2020; Vishnu et al., 2020; Zhang et al., 2020). Compared with the conventional one-stage aging treatment, the two-stage aging (TSA) treatment is considered to be a valid approach to optimize the mechanical performance of most meta-stable β-type Ti alloys. Schmidt et al. (2011) discussed the influence of duplex aging treatment on the fatigue behavior of a typical meta-stable Ti−3Al−8V−6Cr−4Mo−4Zr (wt%). They found that, compared with the one-stage aged specimen, the two-stage aging treatment was employed to improve the fatigue limit value and reduce the rate of fatigue crack growth. The primary reason was that the plenty dispersive ω_iso_ phases result in the uniform precipitation of α phases after the TSA treatment. Cui and Guo (2009) reported the microstructural evolution and tensile performances of a meta-stable Ti−28Nb−13Zr−0.5Fe under various aging conditions. They found that, when the alloy was aged at 350°C, the formation of precipitated α phases would lead to a dramatic increase in the number density of nucleation sites within the β matrix. The isothermal ω transitional phase was transformed into the α phase step by step with the increase in the temperature or the duration of aging, resulting in the refinement of precipitates and the improvement in the strength of alloy at room temperature. Santhosh et al. (2014) proved that the objective of the refinement and the increase in the amount of α phase could be achieved using low-temperature aging. A method of TSA was used to enhance the comprehensive mechanical performances at room temperature. For instance, higher tensile strength and favorable elongation can be achieved using this method. Ivasishin et al. (2008) studied the microstructure control using the different thermo-mechanical working methods in a typical β-type Ti−15V−3Cr−3Sn−3Al alloy. They reported that this alloy with the slowest kinetics in precipitation of the secondary phase could be prepared through the use of duplex aging in order to obtain a relatively higher strength within a limited time for industrial-scale applications.

The Ti−10Mo−6Zr−4Sn−3Nb (Ti-B12) alloy is a newly developed β-Ti alloy used in surgical implants, such as dental archwires and catheter guide wires. It is considered to be an excellent candidate alloy for Ti−6Al−4V, CP-Ti applied in orthopedic surgery and dentistry, owing to its relatively higher tensile strength, lower modulus, and non-toxic alloying elements (Guo et al., 2019; Du et al., 2020, Rabadia et al., 2019). The Ti-B12 alloy is developed by Shaanxi Key Laboratory of Biomedical Metal Materials based on the *d*-electron theoretical approach (Cheng et al., 2020). The room-temperature mechanical performance for the meta-stable β-type Ti-B12 alloy can be controlled and optimized through the use of different solution treatments plus aging treatments. The Ti-B12 alloy subjected to solution treatment followed by aging treatment would possess an attractive combination of room-temperature tensile strength and ductility. Moreover, the molybdenum equivalent of the Ti-B12 alloy is calculated to be about 10.9. Generally, a higher molybdenum equivalent value is one of the necessary conditions for the continuous phase transformation of β → ω → α in meta-stable β-Ti alloys (Li et al., 2015, 2016, 2018). Microstructure and micro-texture evolution in the process of aging treatment can result in a significant change in the mechanical performance of the alloy. Therefore, it is necessary to understand the evolution law of microstructure and texture components during the OSA and TSA treatments. The objective is to clarify the phase transformation mechanism of β → α and β → ω → α under various aging conditions.

The room-temperature and elevated-temperature mechanical performances of meta-stable β-type Ti alloys can be improved by the formation of a large number of finer α precipitates uniformly distributed in the β matrix. The meta-stable ω transitional phase can be considered as a type of effective nucleation site for the precipitation of the α phase during the TSA treatment. The isothermal ω transitional phase can be induced by the diffusion of solute atoms during aging at a relatively lower temperature (300–400°C). To clarify the formation mechanism of the ω phase, the crystal structure, and the elemental diffusion during the phase transformation from an unsymmetrical ω embryo to a symmetrical isothermal ω phase is very important. Nevertheless, almost no investigations have been conducted on the above-mentioned scientific questions, and no research efforts have been made to clarify the atomic-scale structure evolution and elemental diffusion of isothermal ω phase after the low-temperature aging treatment. Consequently, the mechanism of phase transformation from β to ω during the low-temperature aging has not been explained yet.

This research work is carried out to investigate the microstructural evolution of Ti-B12 alloy after the OSA and TSA treatments and its influence on room-temperature mechanical performance. Furthermore, the influence of TSA treatment on the precipitation and the transformation mechanism of the α phase is discussed as well. Cytotoxicity evaluation for Ti-B12 alloy is also carried out using the MTT method.

## Experimental Materials and Methods

The Ti-B12 alloy ingot was produced using a vacuum arc remelting furnace (VAR, 50 kg). The ingot was remelted three times to ensure homogeneity and prevent segregation. In this work, the raw materials for VAR included sponge titanium with small particles (particle size: 0.83–12.7 mm; grade 0), Ti-32Mo master alloy with thin sheet-like, industrial zirconium sponge (Zr-1; particle size: 3–8 mm), and Ti-80Sn and Nb-47Ti master alloy with chips-like. The chemical composition of the Ti-B12 alloy ingot is provided in Table 1. The Ti-B12 cast ingot was homogenized at 1,180°C to improve the metallurgical quality. A 6.3 MN rapid forging press and a GFM (Austria) radial forging machine were used to carry out billet forging and final forging in the temperature range of the β and (α + β) phase regions, respectively. Finally, a 250-type bar rolling mill was employed to manufacture hot-rolled round bars (diameter: 9 mm). Several bars were cut using an electric discharge machine and lathe.

**Table 1:** Chemical composition of Ti-B12 alloy ingot used in the present work (wt%).

**Ti**	**Mo**	**Zr**	**Sn**	**Nb**	**C**	**N**	**O**	**H**	**Fe**
Bal.	10.11	5.71	4.32	2.83	0.024	0.017	0.11	0.0037	0.02

Based on the Ti-Mo phase diagram and ITT diagram, it can be deduced that the isothermal ω transitional phase will precipitate within the parent β phase after low-temperature aging. The low-temperature aging treatment is usually carried out below 400°C. Moreover, the α phase is prone to precipitate in the temperature ranging from 500 to 600°C. In our previous work, the finer α phase tends to precipitate after aging at 510°C for 8 h, which is beneficial for the significant improvement in strength at room temperature. First of all, the Ti-B12 alloy bars were subjected to a solution treatment at 790°C for 1 h, followed by water cooling. Subsequently, two types of aging treatments were carried out by electric furnace in air. One-stage aging was carried out at 510°C for 1, 2, 4, and 8 h, respectively. TSA was firstly performed at 325°C for 2 h and then conducted at 510°C for 0.2, 1, 2, 4, and 8 h, respectively.

The microstructure of each specimen was observed using optical microscopy (OM, OLYMPUS BX61), scanning electron microscopy (SEM, FEI Quanta 650F) equipped with electron backscattered diffraction (EBSD, Oxford Instruments + HKL Channel 5 software package), and transmission electron microscopy (TEM) (FEI Tecnai G2 F20). EBSD and X-ray diffraction (XRD, Bruker D8 Focus) techniques were used to characterize the evolution of microstructure and crystallographic orientation. The polished bulk specimen was examined by XRD to analyze the phase constituent and phase transformation. The measurements of XRD were carried out in the range of 30–80°, with a constant step of 0.02° and a scanning speed of 6°/min. The accelerating voltage and current are 40 kV and 40 mA, respectively.

Uniaxial tensile testing was conducted using an INSTRON 598X system (maximum load: 250 kN) at room temperature. The strain rate was ~0.006 mm/min. Tensile specimens were machined following the GB/T 228-2007 standard. The diameter and the gauge length of the tensile samples were 3 and 15 mm, respectively. The specimens were etched using Kroll reagent (8% HNO_3_ + 2% HF + 90% H_2_O, vol%) for 5–10 s. The fracture surface and the microstructure of the specimen were characterized by SEM in secondary electron mode. TEM observation was conducted by a FEI Tecnai G2 F20 operated at an accelerating voltage of 300 kV. TEM specimen was mechanically ground to a foil with a thickness of 40 μm using SiC paper until 2000 grade. The foil was punched into several discs of 3 mm in diameter, which would be thinned by the ion milling technique (Gatan 691) for the TEM characterization.

MTT test was carried out to evaluate the cytotoxicity and biocompatibility of the implanted material using L929 cells. GB/T 16886.5-2017 biological evaluation of medical devices—part 5: tests for *in vitro* cytotoxicity was used in this work. The L929 cells in the logarithmic growth phase were selected to be digested with trypsin. The cell culture medium was diluted to a L929 cell suspension with a concentration of 1 × 10^4^/ml. They were inoculated on four 96-well culture plates (0.20 ml cell suspension per well). The cells were cultured for 24 h at 37°C. The original culture medium was discarded after the cells adhered to the wall. After rinsing with physiological saline three times, two kinds of extracts for Ti-B12 and Ti−6Al−4V alloy and RPMI1640 cell culture medium were added to a 48-well plate (0.20 ml cell suspension per well). They were incubated at 5% CO_2_ atmosphere at a constant temperature (37°C). The respective culture solution was carefully replaced after 72 and 120 h. A culture plate was taken out, and the original culture medium was carefully aspirated after 24, 72, 120, and 168 h. The 0.20 ml fresh RPMI1640 culture medium and 20 μl MTT (5 mg/ml, thiazolyl blue) solution were added to the culture plate and continued to be cultured for 4 h. The solution in wells was carefully removed and washed twice with physiological saline; 0.20 ml dimethyl sulfoxide (DMSO) was added into each well. The culture plate was gently shaken for 10 min, and the absorbance value (OD value) of each well with an enzyme-linked immunoassay at 490-nm wavelength was measured using 0.20 ml DMSO as a reference.

## Results and Discussion

### Microstructure for Solution-Treated, One-Stage-Aged, and Two-Stage-Aged Specimens

**Figure 1:**
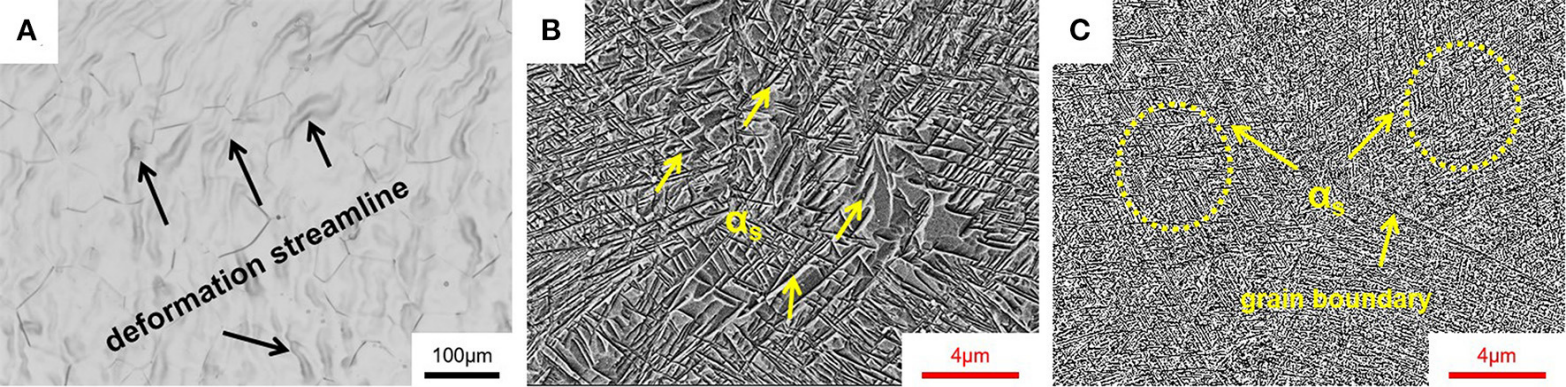
Microstructures of Ti-B12 alloy subjected to **(A)** solution treatment at 790°C for 1 h followed by air cooling (optical microscopy), **(B)** one-stage aging at 510°C for 4 h after solution treatment (scanning electron microscopy, SEM), and **(C)** two-stage aging (TSA) at 325°C for 2 h followed by 510°C for 4 h after solution treatment (SEM).

Figure 1 displays OM and SEM images for Ti-B12 alloy subjected to solution-treated, one-stage aging, and two-stage aging treatments. Based on previous investigations (Rack et al., 1970; Laheurte et al., 2006; Cai et al., 2012, 2013), the Ti-B12 alloy subjected to an appropriate solution treatment in conjunction with aging treatment could achieve outstanding comprehensive mechanical properties. Therefore, in this work, based on β transus temperature (*T*_β_ = 760°C) measured using the conventional metallographic procedure, the solution heat treatment is determined to be 790°C/h and air cooling (AC). As shown in Figure 1A, the specimen subjected to solution treatment followed by air cooling is mainly composed of equiaxed β grains. Based on the analysis by Image pro-plus 6.0 software, the average grain size is about 88 μm. Some deformation streamlines also exist in the microstructure. Figure 1B displays the SEM image for the Ti-B12 alloy bar subjected to the OSA. Cluster-like α phase with micron-scale was precipitated in the β grains of the sample subjected to the OSA for 4 h. The SEM image for the Ti-B12 alloy subjected to solution treatment in conjunction with low-temperature aging at 325°C for 2 h followed by high-temperature aging at 510°C for 4 h is presented in Figure 1C. It can be clearly seen that the α phase continuously precipitates at the grain boundary triple junctions of β grains. Meanwhile, a large amount of fine and dispersive α phases with “needle-like” shape are generated within the β equiaxed grains. Compared with Figures 1B,C shows that the size of the α phase precipitating in the sample subjected to TSA is significantly finer than that subjected to OSA. It is noteworthy that there are some differences between the nucleation site and the growth rate of grains under various heat treatments.

**Figure 2:**
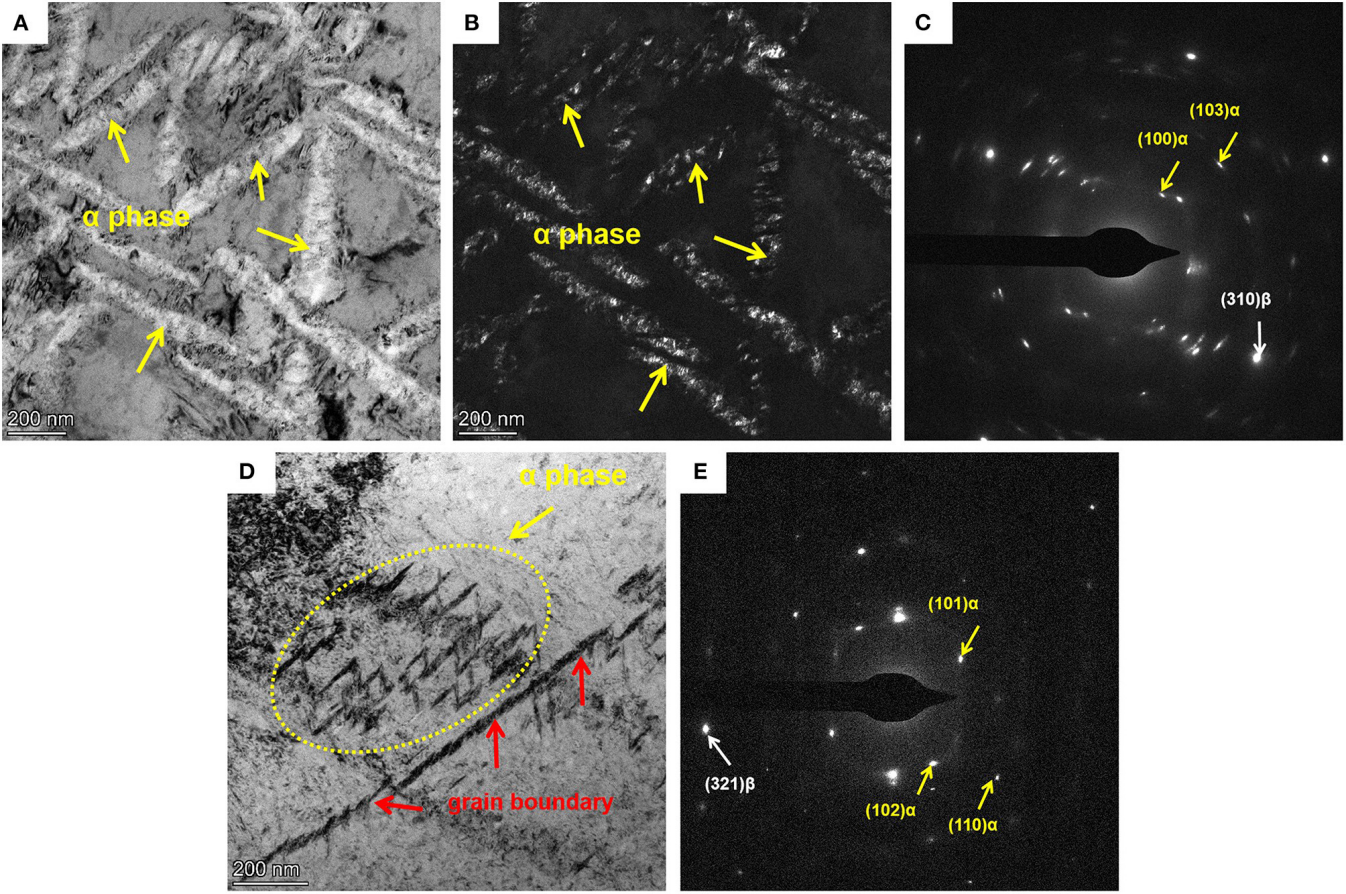
Transmission electron microscopy (TEM) pictures for Ti-B12 alloy subjected to one-stage aging (OSA) and two-stage aging (TSA) **(B,C)** OSA at 510°C for 1 h **(D,E)** and TSA at 325°C for 2 h followed by 510°C for 1 h. **(A,D)** TEM bright-field images, **(B)** TEM dark-field images, and **(C,E)** selected area diffraction patterns.

The TEM bright-field (BF) images and the corresponding selected area diffraction (SAD) patterns of Ti-B12 alloys subjected to OSA at 510°C for 1 h and TSA at 325°C for 2 h followed by 510°C for 1 h are presented in Figure 2. In general, BF and dark-field (DF) images are employed to characterize the morphology and distribution of secondary phase in the metallic materials. Meanwhile, SAD patterns are often used to illustrate the phase transition mechanism and orientation relationship after various aging treatments. Figure 2C presents a SAD pattern of [001]β zone axis for Ti-B12 alloy subjected to solution treatment followed by OSA. It can be found that the reflection spots are presented at 1/2 {211}β positions, which reveals that the α phase precipitates from the parent β phase after aging. As presented in Figures 2A,B, te α precipitates in the OSA specimen are needle-shaped. The length is ~0.675 μm, and the width is about 0.087 μm. The morphologies of the α phases precipitated from the β matrix after low-temperature aging at 325°C for 2 h followed by high-temperature aging at 510°C for 1 h are shown in Figure 2D. It can be found that the refining effect of the α phase in the TSA specimen is obvious compared to the OSA one. The morphology of the α phases precipitated from the β matrix after TSA is short shuttle-like. The length and width are about 0.123 and 0.0228 μm, respectively. As seen in the difference between Figures 2A,D, it can be noticed that the α phase has been refined obviously after the TSA treatment, owing to the effect of the isothermal ω transitional phase-assisted phase transition. Furthermore, as seen in Figure 2E, the SAD pattern of the Ti-B12 alloy does not display any additional reflection spots because a large number of isothermal ω transitional phases precipitated from the parent β phase during the low-temperature aging treatment have completely transformed into α precipitates after the high-temperature aging at 510°C for 1 h. As can be observed in Figures 2B,D, it is necessary to note that the isothermal ω transitional phase-assisted nucleation mechanism plays a significant role in the precipitation and growth of the precipitated α phase within the β grains. However, the accurate nucleation positions of the α precipitates are too difficult to be confirmed only by TEM dark-field images (Nag et al., 2009; Zheng et al., 2016a,b; Chen et al., 2018; Shi et al., 2019).

### Phase Transition in Ti-B12 Alloy After the Two-Stage Aging Treatment

**Figure 3:**
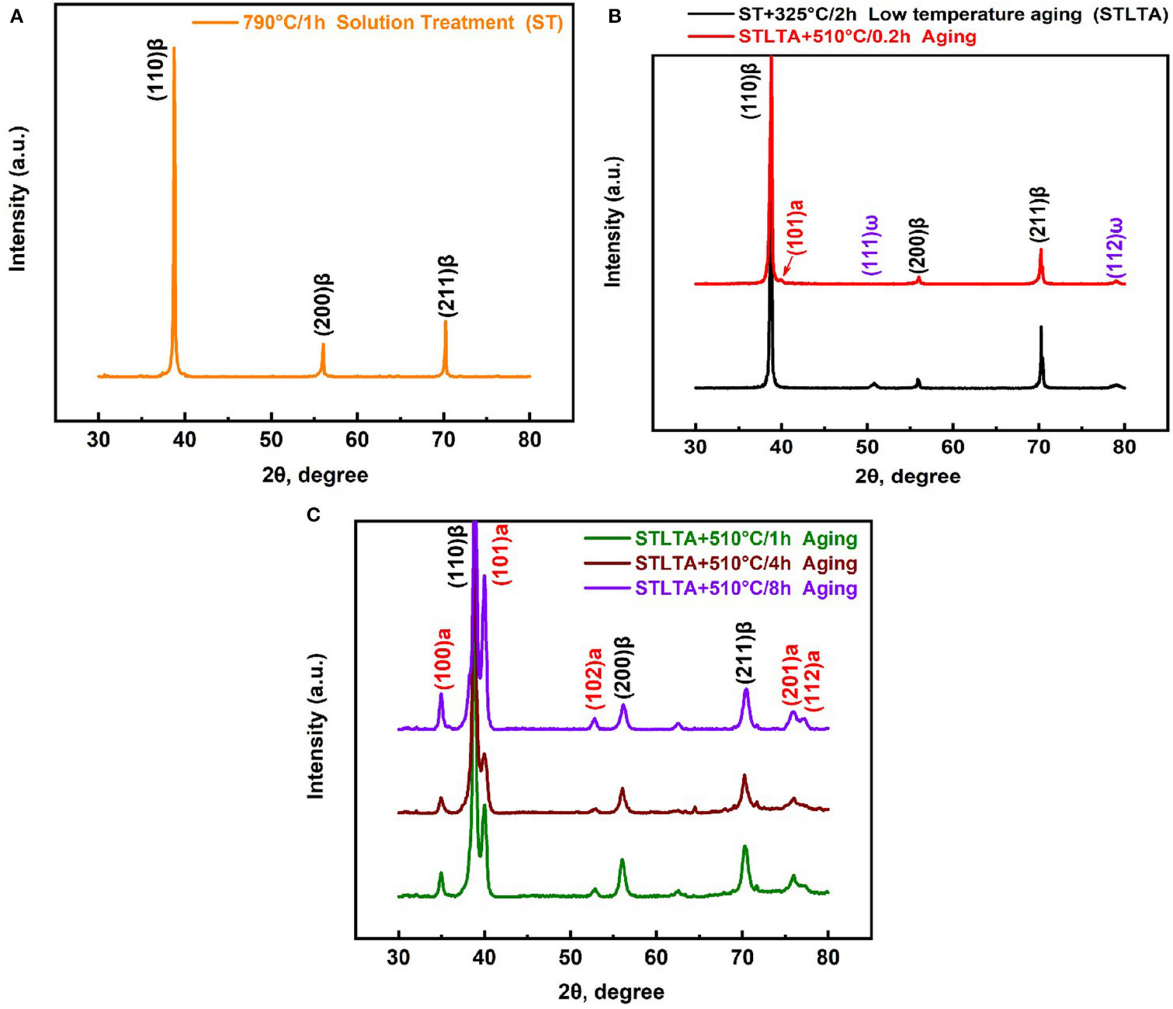
X-ray diffraction patterns for Ti-B12 alloy subjected to solution treatment **(A)**, solution treatment followed by low-temperature aging (LTA) at 325°C for 2 h **(B)**, and LTA followed by high-temperature aging at 510°C for 0.2 h **(B)** and 1, 4, and 8 h **(C)**.

**Figure 4:**
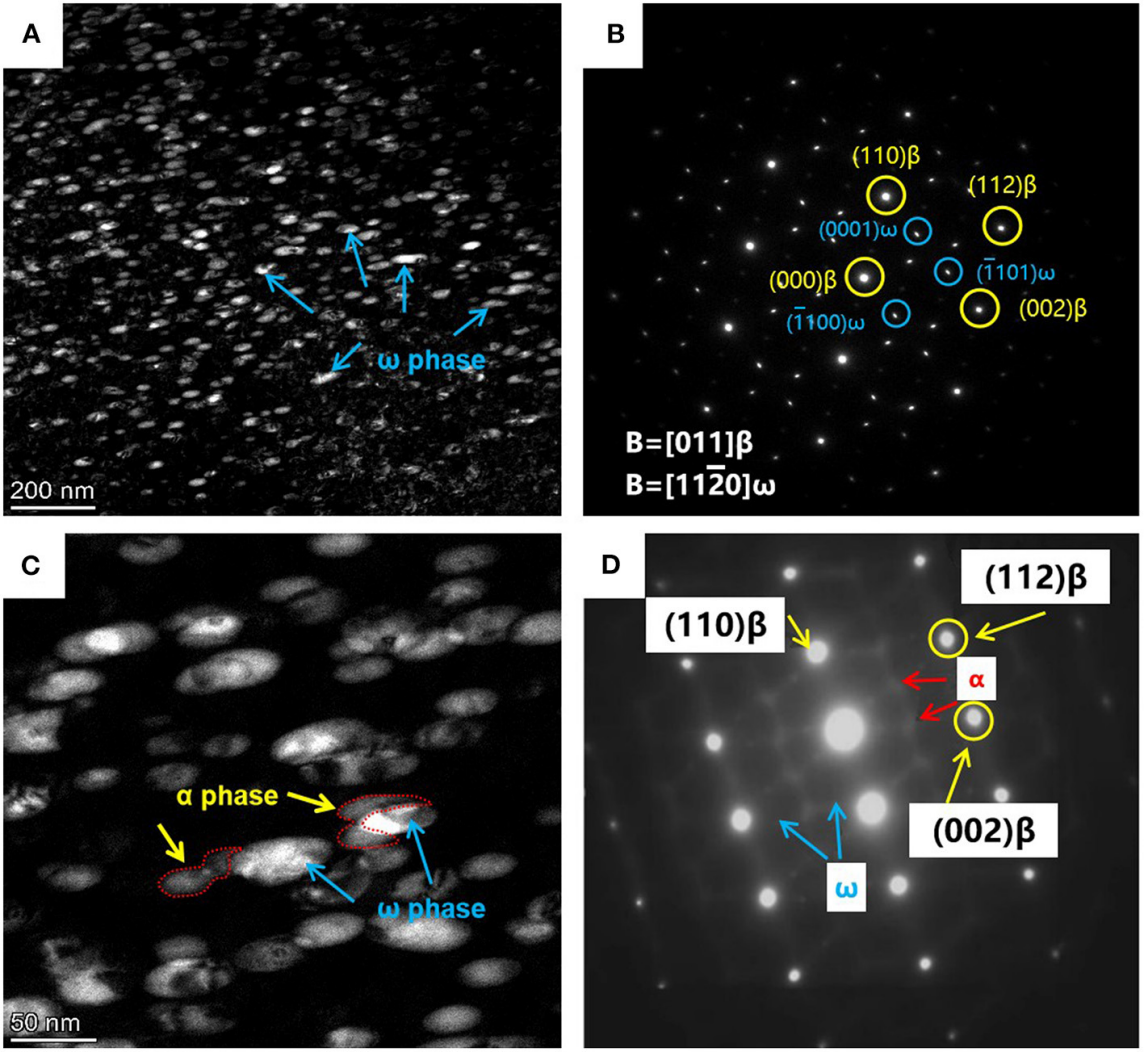
Transmission electron microscopy (TEM) pictures for Ti-B12 alloy solution treated at 790°C for 1 h plus **(A,B)** low temperature aging at 325°C for 2 h and **(C,D)** low-temperature aging at 325°C for 2 h followed by high-temperature aging at 510°C for 0.2 h. **(A,C)** TEM dark-field images. **(B,D)** Selected area diffraction patterns.

The XRD patterns for the Ti-B12 alloy subjected to solution treatment at 790°C in conjunction with low-temperature aging at 325°C followed by high-temperature aging at 510°C are shown in Figure 3. The XRD spectra for the Ti-B12 specimen solution-treated at 790°C for 1 h followed by water quenching are shown in Figure 3A. The experimental result indicates that there are some diffraction peaks of the β phase with a body-centered cubic structure because the solution temperature is located in the temperature range of the β phase zone. As can be seen in Figure 3B, the diffraction peaks of the ω phase begin to emerge in (111) and (112) planes during low-temperature aging at 325°C for 2 h. The co-existence of the diffraction peaks for β and ω phases indicates that the phase transition from ω phase to α phase has not yet taken place after low-temperature aging. Furthermore, the SAD pattern for the low-temperature aged specimen is presented in Figure 4B. It can be deduced that there are no α precipitates which are transformed from the β parent phase or isothermal ω transitional phase under this condition. When the Ti-B12 alloy was subjected to low-temperature aging at 325°C for 2 h followed by high-temperature aging at 510°C for 0.2 h, the (101)α diffraction peak is presented. This phase transformation is also accompanied by the appearance of isothermal (111)ω and (112)ω diffraction peaks. Moreover, when the Ti-B12 alloy is subjected to low-temperature aging at 325°C for 2 h followed by high-temperature aging at 510°C for 0.2 h, the precipitated α phase with finer scale would be transformed from isothermal ω transitional phase or β parent phase. Previous investigations showed that the phase transition from ω phase to α phase would be induced in some biomedical β Ti alloys (Chaves et al., 2015; Wang et al., 2018). As can be seen in Figure 3C, the XRD results for the TSA specimen (low-temperature aging at 325°C for 2 h followed by high-temperature aging at 510°C for 1 h) show that there is no isothermal ω transitional phase retained after the high-temperature aging at 510°C for 1 h. The result of the XRD is identical to that of the SAD pattern for the Ti-B12 alloy in Figure 2E. Moreover, it can be seen in Figure 3C that extending the aging time will result in the enhancement of (100)α, (101)α, and (102)α diffraction peak intensity in the XRD spectrum. Compared with the XRD spectra of the Ti-B12 alloy subjected to aging at 510°C for 0.2 and 1 h, the results of the XRD spectra for the Ti-B12 specimens subjected to aging at 510°C for 4 and 8 h indicate that the quantity of α precipitates obviously increases with the prolongation of aging time. Meanwhile, the intensity of (100)α, (101)α, and (102)α diffraction peaks also increases under the above-mentioned heat treatment conditions.

Figures 4A,B present the DF image and SAD pattern of the Ti-B12 alloy subjected to low-temperature aging at 325°C for 2 h. As can be observed in Figure 4A, a lot of ellipsoid-like isothermal ω transitional phases with the size of ~57 nm are precipitated from the parent β phases. As shown in Figure 4B, reflection spots are presented at 1/3 and 2/3 {211}β positions of the Ti-B12 alloy subjected to solution heat treatment at 790°C for 1 h plus low-temperature aging at 325°C for 2 h, owing to the precipitation of ω phases. The result of Figure 4B will coincide with the specific orientation relationship of [210](002)β // [−1011](−11-1)ω1 // [−2111](−101)ω2 (Qazi et al., 2005; Cui and Guo, 2009; Nag et al., 2009; Wang et al., 2009). As can be seen in Figure 4C, there is evidence that the short shuttle-like α phases precipitate with the assistance of the isothermal ω phase during aging treatment. The enlarged image provides a strong evidence for the evolution of the ω transitional phase transformed into α phase. It can be reasonably deduced that the potential nucleation and growth of a new phase may emerge at the boundaries between parent β phase and ω_iso_ phase. Previous research work has been carried out for the investigation of phase transformation in Ti−5Al−5Mo−5V−3Cr−0.5Fe (Ti-5553) alloy (Nag et al., 2009). A SAD pattern of the Ti-B12 alloy subjected to solution treatment followed by low-temperature aging at 325°C for 2 h and high-temperature aging at 510°C for 0.2 h is presented in Figure 4D. Reflection spots are presented at 1/3 and 2/3 {110}β positions because of the existence of a residual isothermal ω transitional phase during the low-aging treatment. Moreover, the emergence of newly generated reflection spots corresponding to the α phase demonstrates that these α precipitates are transformed from the isothermal ω transitional phase or the parent β phase during high-temperature aging treatment at 510°C for 0.2 h.

### Texture Evolution of Ti-B12 Alloy Subjected to OSA and TSA

**Figure 5:**
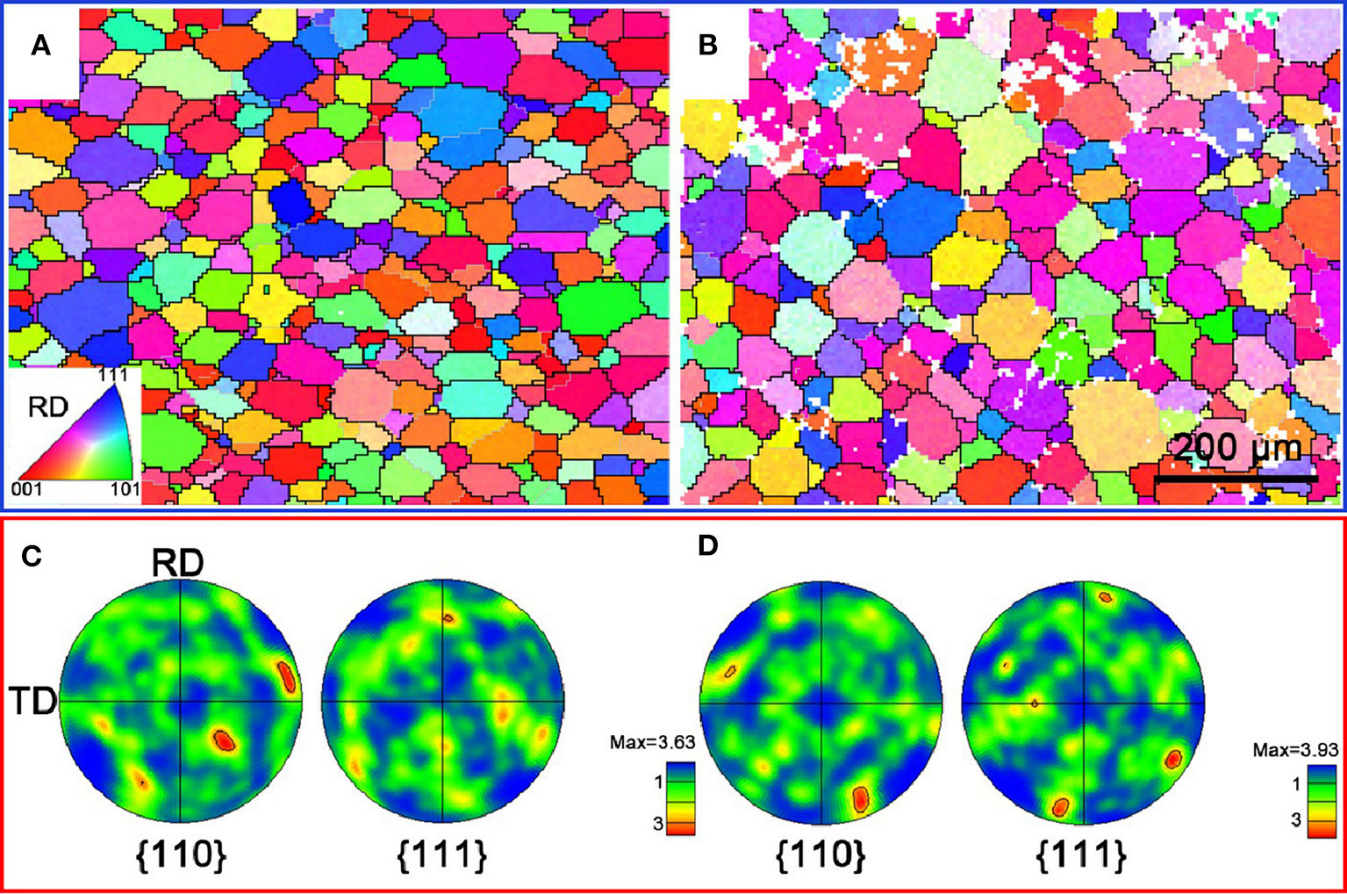
Electron backscattered diffraction maps of Ti-B12 alloys subjected to one-stage aging (790°C/h, AC + 510°C/8 h, AC) and two-stage aging (790°C/h, AC + 325°C/2 h, AC + 510°C/8 h, AC). **(A,B)** Inverse pole figure maps. **(C,D)** Pole figure maps.

EBSD maps of Ti-B12 alloys subjected to one-stage aging and two-stage aging are shown in Figure 5. It can be seen from the inverse pole figure (IPF) maps (Figures 5A,B) that complete recrystallization can be obtained in the alloy after solution treatment followed by aging. Meanwhile, the β grain size of the TSA sample is slightly larger than that of the OSA sample. As can be observed in Figures 5C,D, the β grain orientation of the Ti-B12 alloys subjected to OSA and TSA presents a random distribution. The maximum values of pole density are 3.63 and 3.93, respectively.

**Figure 6:**
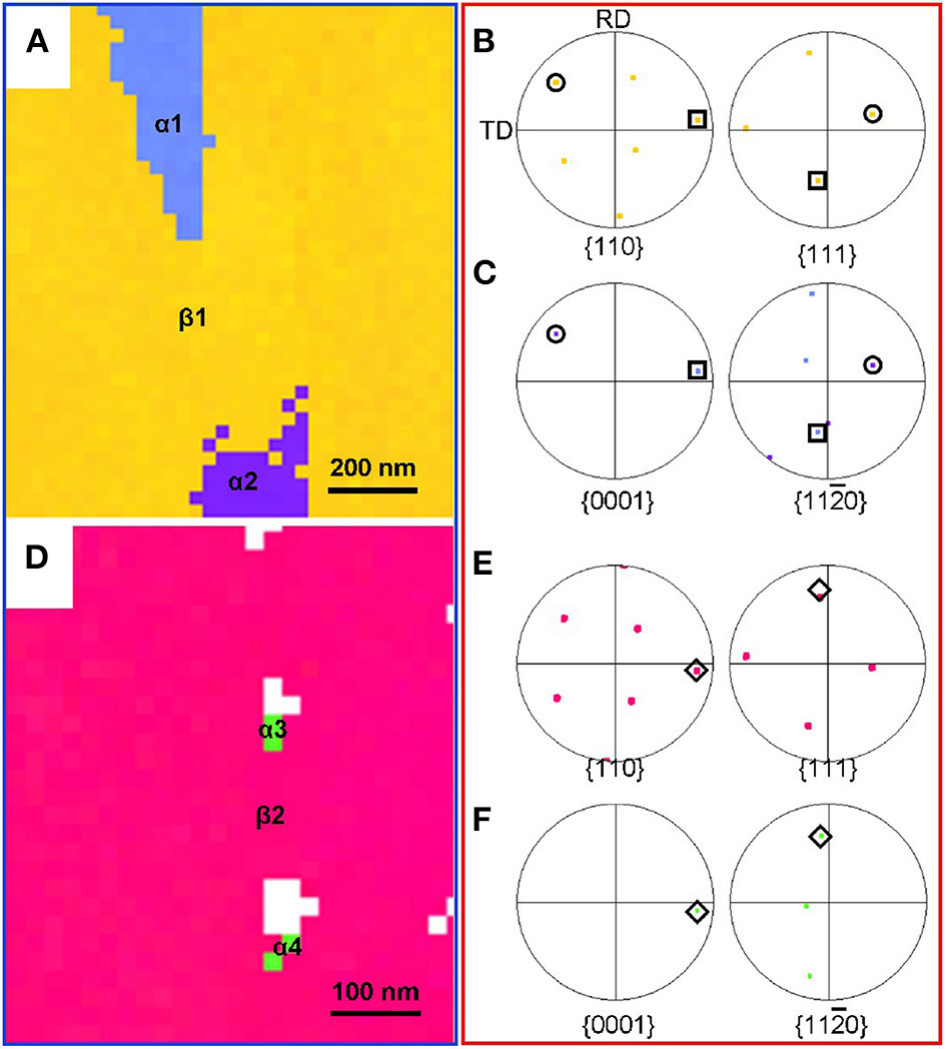
Results of the orientation relationship between α and β phases. **(A–C)** Oone-stage aging (790°C/h, AC + 510°C/8 h, AC) condition, **(D–F)** two-stage aging (790°C/h, AC + 325°C/2 h, AC + 510°C/8 h, AC) condition. **(A,D)** Inverse pole figure maps. **(B,C,E,F)** Pole figure maps.

A small step (20 nm) is applied to collect the crystal orientation information on the α and β phases in order to clearly characterize the orientation relationship between the parent phase and the precipitated phase. The results of the orientation relationship between the α and β phases are displayed in Figure 6. Figure 6A presents the IPF map of the OSA sample, which contains one β grain and two α grains (α1 and α2). Figures 6B,C are the pole figures of the β grains and the α phases, respectively. It can be clearly seen that the α1, α2, and β1 grains satisfy the following orientation relationship: {0001}α // {110}β and <11-20>α // <111>β, that is to say, the β → α in the OSA specimen follows the typical Burgers orientation relationship (BOR) (Chai et al., 2017, 2018). Similarly, Figure 6D shows the IPF map of the TSA specimen. As can be seen in Figures 6E,F, both α and β phases follow the BOR as well.

Previous investigations have indicated that the finer α precipitates are formed by contact with the surface of the isothermal ω phase. The habit plane is usually defined as (11-20) ω // (11-20) α. It is proved that the α phase tends to nucleate at the interface between the ω and β phases. The reason is that this interface is regarded as the minimum misfit low-index habit plane between ω and β phases, resulting in a significant reduction of apparent activation energy for the nucleation and growth of α precipitates (Ohmori et al., 2001; Cremasco et al., 2011; Chen et al., 2019; Dong et al., 2020; Xiang et al., 2020). Hence, α phases with the aid of the isothermal ω phase-assisted nucleation will possess a basically similar BOR with those formed under the OSA condition. It can also be reasonably deduced that, although the size of the secondary phase has become obviously smaller, the phase transition from β to α phase with the aid of isothermal ω phase cannot change the crystallographic orientation of the precipitated phase.

### Mechanical Properties at Room Temperature and Fracture Behavior for OSA and TSA Ti-B12 Alloy

**Figure 7:**
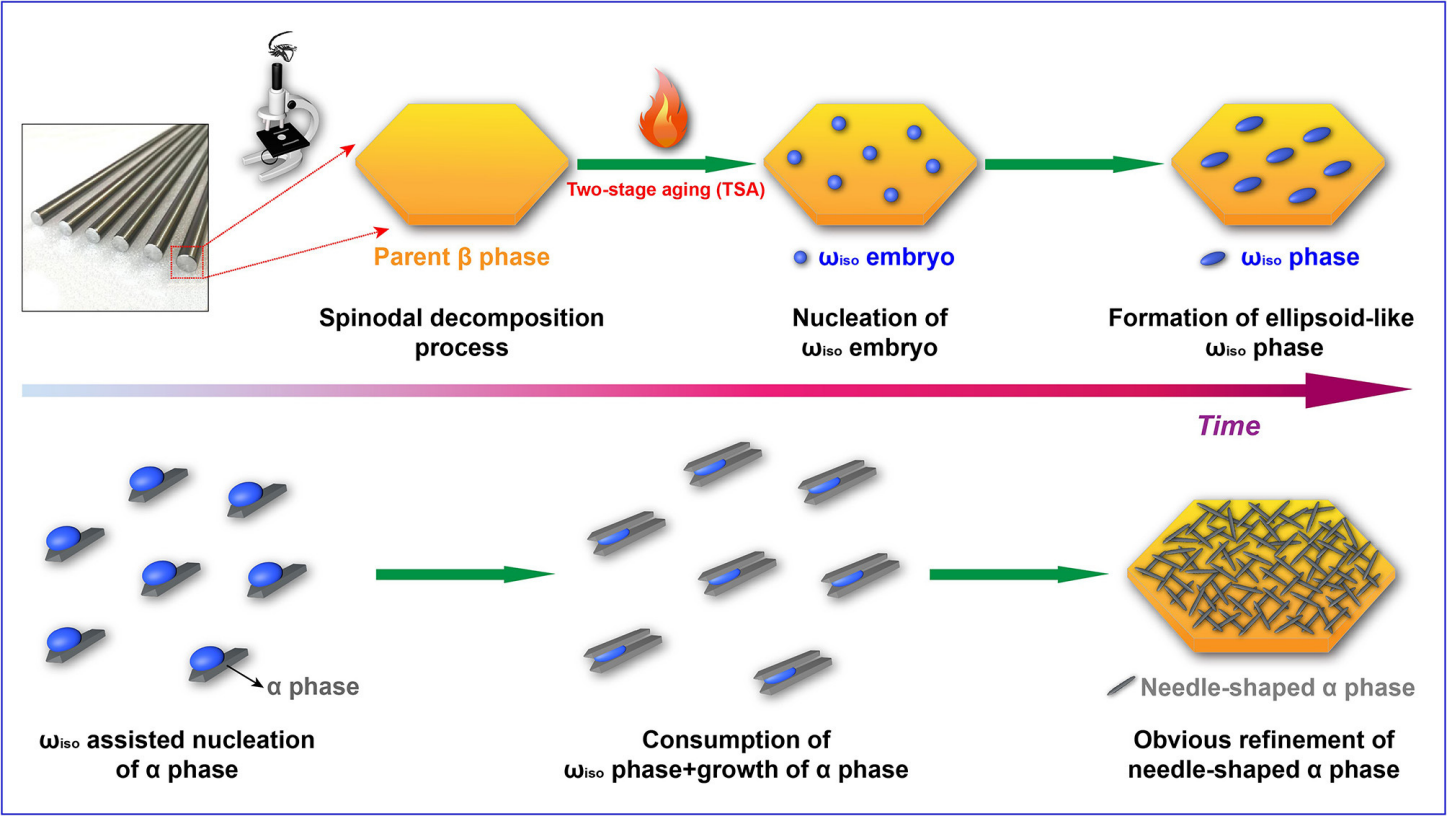
Schematic drawing of the isothermal ω transitional phase-assisted phase transformation from β to α.

The most outstanding mechanical properties for the Ti-B12 alloy can be achieved after TSA (low-temperature aging followed by high-temperature aging). An attractive matching degree of strength and elongation can be obtained through the use of low-temperature aging at 325°C for 2 h followed by high-temperature aging at 510°C for 8 h. In this case, the influence of low-temperature aging at 325°C on the microstructural evolution is favorable for the refinement of α precipitates. The reason for this phenomenon is that the isothermal ω transitional phase precipitated during low-temperature aging will result in the increase in the density of nucleation sites for the phase transformation from β phase to α phase. Therefore, the adoption of TSA is beneficial to form a large number of dispersed α precipitates with a significantly small size in the β matrix. A schematic drawing of isothermal ω transitional phase-assisted phase transformation from β to α is presented in Figure 7. In general, the process of isothermal ω transitional phase-assisted α precipitates predominantly includes the nucleation, multiplication, growth of ω embryo in conjunction with precipitation and, near *in situ* growth of α phase as well as the consumption of ω precipitates during the whole aging process. Both the precipitation of the ω phase and the parent β phase satisfy a given crystallographic orientation relationship, that is, the habit plane. The growth orientation of the isothermal ω phase is considered to be in the invariant direction of the habit plane. Moreover, there is a Burgers vector whose constant normal is perpendicular to the habit plane. The crystallographic orientation relationship of constant normal is maintained unchanged during the whole phase transformation process. Assuming that the constant normal in the real space and the one in the reciprocal space are all determined, the growth direction of the precipitated phase can be deduced based on the orientation relationship between the ω and β phases. The ω phase possesses a {111}β habit plane, and the growth direction of the precipitated phase is usually <14 14 15>β. The migration interface between the ω and α phases is extremely close to the habit plane of the α phase, and the deviation angle of the two planes is about 5.1°. Since the Ti-B12 alloy is subjected to low-temperature aging, the morphological characteristics of the ω phase are strongly influenced by the role of the secondary α phase nucleation and growth, owing to the intimate contact between the isothermal ω precipitated phase and the parent β phase as well as the anisotropy of the α precipitates. The α precipitates are prone to nucleate at the ω/β interface. In the initial stage of low-temperature aging, the isothermal ω phase is quickly exhausted by the secondary α phase, and it finally disappeared under the influence of ω-assisted α phase precipitates. Consequently, the initial ellipsoid-like ω phase will be decomposed along the direction of the ω/α interface migration, leading to the formation of a short shuttle-like precipitated phase during TSA.

**Figure 8:**
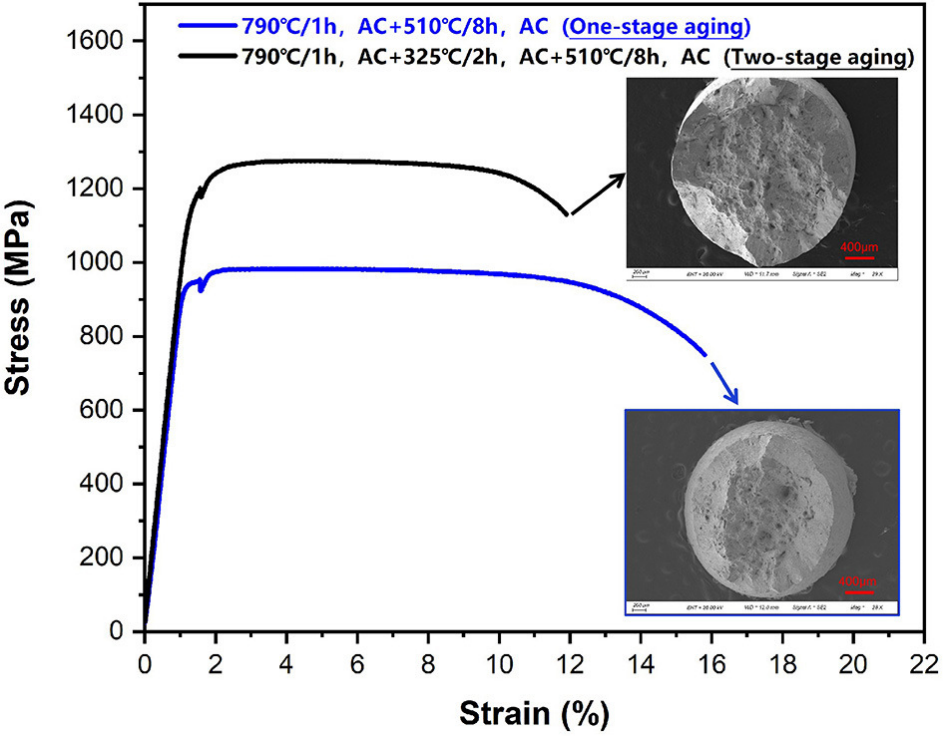
Tensile curves (engineering stress–engineering strain) for Ti-B12 alloy subjected to one-stage aging (790°C/h, AC + 510°C/8 h) and two-stage aging (low-temperature aging at 325°C for 2 h followed by high-temperature aging at 510°C/8 h) after solution treatment (790°C/h, AC). The inset shows the corresponding fracture surface at low magnification.

The tensile curves of the Ti-B12 alloy subjected to OSA (510°C for 8 h) and TSA (low-temperature aging at 325°C for 2 h followed by high-temperature aging at 510°C for 8 h) after solution treatment are presented in Figure 8. In contrast to the OSA specimen, a relatively higher strength and an acceptable elongation at room temperature can be obtained through the TSA process. As can be seen in Figure 8, the tensile strength of the OSA specimen subjected to aging at 510°C for 8 h is about 982 MPa. However, the most outstanding mechanical properties (tensile strength: 1,275 MPa, elongation: 12%) can be achieved in the TSA Ti-B12 alloy, owing to its peculiar microstructure. In addition, a serration phenomenon can be observed in the stress–strain curve. Such a serration phenomenon is attributed to the effect of stress relaxation during tensile testing at room temperature. Therefore, this phenomenon is inevitable. The microstructure and fracture behavior for the alloy will be described in detail in the following paragraphs.

**Figure 9:**
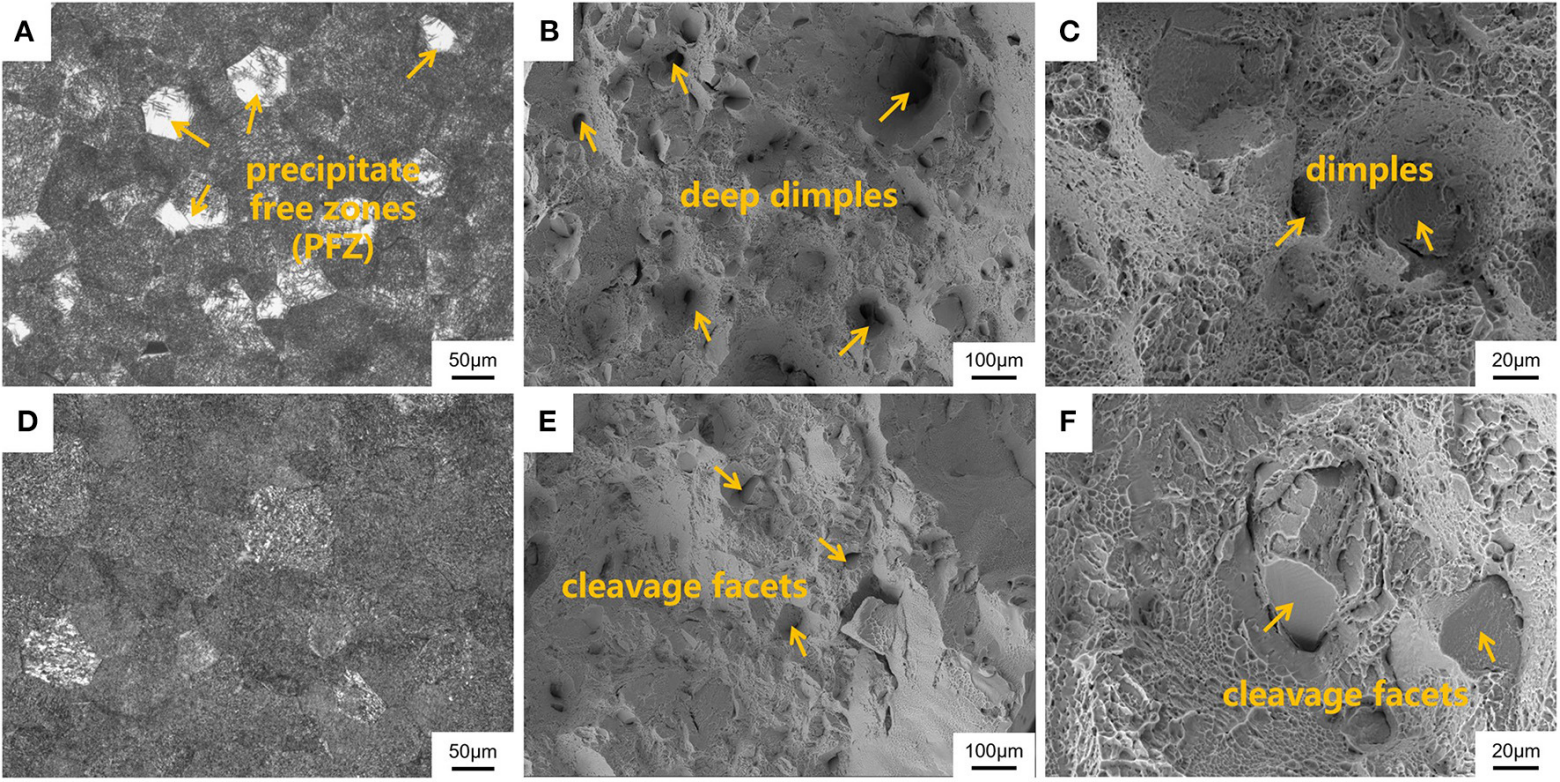
Scanning electron microscopy images of the microstructures and fracture surfaces for Ti-B12 alloy subjected to **(A–C)** solution treatment (790°C/h, AC) plus one-stage aging treatment at 510°C for 8 h and **(D–F)** solution treatment (790°C/h, AC) in conjunction with two-stage aging treatment at 325°C for 2 h followed by 510°C for 8 h. **(A,D)** Optical microstructures. **(B,C,E,F)** Magnified images of fracture surfaces.

The mechanical properties for some β-type Ti alloys are greatly affected by the precipitation of secondary phases after aging treatment because the mechanical properties, including strength and ductility, strongly depend on the density (ρ) and volume fraction (vol.%) of the precipitated phases within the parent phase (Gao et al., 2019; Yang et al., 2019; Li et al., 2020; Tang et al., 2020; Xiao et al., 2020; Lai et al., 2021). Hence, optical microscopy can be employed to analyze and evaluate the peculiar microstructure for various aged specimens and the plastic deformation behavior. The optical images for the microstructures for OSA (790°C/h, AC + 510°C/8 h, AC) and TSA (low-temperature aging at 325°C for 2 h followed by high-temperature aging at 510°C for 8 h) specimens are presented in Figures 9A,D. It can be seen in Figure 9A that there are some precipitate-free zones existing in the microstructure and continuously thick α layers covering a portion of grain boundaries entirely in the OSA sample. Therefore, we can suppose that low-temperature aging is critical to increase the number of α precipitates and inhibit the coarsening of α layers. In contrast, as can be observed in Figure 9D, the α layers and the size of α phases within the β grains for the TSA specimen are obviously smaller than those of the OSA one. Meanwhile, as can be seen in Figure 1C, the region around triple grain boundaries displays the morphology of the α phase precipitated within the β grains and continuous α layers in the boundaries at high magnification. A large number of isothermal ω transitional phases can be regarded as effective obstacles that prevent the emergence of α layers in the boundaries and the growth of secondary phase within the β grains. Figures 9B,C,E,F show the morphologies of the fracture surfaces at low and high magnification in the OSA and TSA samples. It can be seen from the fracture surfaces of the OSA and TSA samples that the predominant mechanism is the ductile fracture with a certain degree of cleavage fracture feature after tensile deformation at room temperature. As can be seen in Figure 9B, transgranular fracture and some deep dimples are observed on the fracture surface of the Ti-B12 alloy after OSA. As shown in Figure 9C, some evidence of predominantly ductile and slightly brittle fracture is detected on the fracture surface using secondary electron mode. Therefore, it can be deduced that a mixed fracture behavior is exhibited in the Ti-B12 alloy subjected to OSA at 510°C for 8 h after solution treatment in the temperature range of the β phase region. In particular, a transgranular fracture mode plays a more dominant role in the alloy after tensile deformation to some extent. Moreover, Figures 9E,F indicate that intergranular fracture plays a pivotal role during tensile deformation. The numbers of dimples decrease sharply and become shallow. The overall number of cleavage facets in the fracture surface increases obviously after tensile deformation at room temperature. The scale of dimples is ~40–90 μm. This characteristic is associated with the shape of the α phase, owing to the refinement of the secondary phase and the absence of thick α layers in the boundaries. This comparative study on the fracture behavior of the Ti-B12 alloy can demonstrate the negative influence of the thick α layers in the grain boundaries on the elongation and the positive role of TSA on the fracture behavior at room temperature. Hence, one can conclude that the employment of TSA can effectively prevent the coarsening of the α layers in the boundaries to achieve an attractive combination of higher room-temperature tensile strength and reasonable ductility.

### Cytotoxicity Evaluation for Ti-B12 Alloy Using the MTT Method

The relative number of L929 cells in each well (indicated by the OD value) is determined using the MTT method after 24, 72, 120, and 168 h. The measured OD values are displayed in Table 2; relative rate of cell proliferation = (OD value of experimental group / OD value of blank control group) × 100%. The relative cell proliferation rate is calculated. The evaluation criteria are obtained for the cytotoxicity of implanted material. The relative proliferation rate of cytotoxicity levels 0, 1, 2, 3, and 4 is ≥100, 80–99, 50–79, 30–49, and 0–29%, respectively. The results of the cytotoxicity test indicate that, compared with the cell culture medium, neither the Ti-B12 nor the Ti−6Al−4V alloy showed obvious cytotoxicity. The Ti−6Al−4V alloy has been widely used as an implanted material. In addition, it can be seen that the cytotoxicity of the Ti-B12 alloy is slightly better than that of the Ti−6Al−4V alloy, which can meet the requirements of medical materials for material cytotoxicity. Thus, it is evident that the Ti-B12 alloy has excellent biocompatibility.

**Table 2:** The result of cytotoxicity measured by MTT assay (OD values) (*n* = 24).

**Time (h)**	**OD values**		
	**RPMI1640**	**Ti-B12 (relative proliferation rate, %)**	**TC4 (relative proliferation rate, %)**
24	0.235 ± 0.021	0.240 ± 0.018 (102)	0.230 ± 0.027 (98)
72	0.532 ± 0.026	0.528 ± 0.025 (99)	0.515 ± 0.028 (97)
120	0.894 ± 0.035	0.887 ± 0.032 (99)	0.850 ± 0.042 (95)
168	1.15 ± 0.042	1.12 ± 0.038 (97)	1.10 ± 0.051 (96)

## Conclusions

The influence of isothermal ω transitional phase-assisted phase transition for the Ti−10Mo−6Zr−4Sn−3Nb (Ti-B12) alloy is investigated in this work. The microstructural evolution and mechanical performance at room temperature of the Ti-B12 alloy subjected to OSA and TSA treatment are investigated using OM, SEM, TEM (SAD), XRD, and EBSD. MTT test was carried out to evaluate the acute cytotoxicity and biocompatibility of the implanted material. The main conclusions can be summarized as follows:

1. The small ellipsoid-like isothermal ω transitional phases with the size of ~57 nm precipitated from the parent β phase when the Ti-B12 alloy is subjected to solution treatment at 790°C for 1 h and air cooling in conjunction with low-temperature aging at 325°C for 2 h. A definite orientation relationship (OR) is presented between the ω phase and the β matrix. While the alloy is subjected to high-temperature aging treatment at 510°C, a large number of short shuttle-like α phases precipitate at positions of the isothermal ω transitional phases. Such a microstructure is considered to be favorable for the room-temperature mechanical properties of the Ti-B12 alloy.
2. The Ti-B12 alloy subjected to OSA at 510°C for 8 h possesses a relatively lower tensile strength, owing to the coarsening of the α phases on the β grain boundaries and in the β grains. The precipitated α phases in the OSA alloy are directly generated in the parent β phase, which fulfills the BOR of (0001)α // (110)β. In addition, the crystallographic orientation of the α phases in the TSA alloy subjected to low-temperature aging at 325°C for 2 h followed by high-temperature aging at 510°C for 8 h is basically analogous to those in the OSA due to the existence of (10–20)ω // (10–20)α orientation relationship after the high-temperature aging.
3. The Ti-B12 alloy subjected to TSA (low-temperature aging at 325°C for 2 h followed by high-temperature aging at 510°C for 8 h) possesses outstanding comprehensive mechanical properties (tensile strength: 1,275 MPa, elongation: 12%) at room temperature. A large number of isothermal ω transitional phases can be regarded as effective obstacles that prevent the emergence of thick α layers in boundaries and the rapid growth of secondary phases in the β grains for the Ti-B12 alloy subjected to low-temperature aging at 325°C. The morphologies of the fracture surfaces in the TSA specimen show that the number of dimples tends to decrease and become shallow; the intergranular fracture mechanism plays a major role during tensile deformation.
4. The relative proliferation rate of cytotoxicity levels 0, 1, 2, 3, and 4 is ≥100, 80–99, 50–79, 30–49, and 0–29%, respectively, for the Ti-B12 alloy. The cytotoxicity of the Ti-B12 alloy is slightly better than that of the Ti−6Al−4V alloy, which can meet the requirements of biomedical materials.

## Data Availability Statement

The original contributions presented in the study are included in the article/supplementary materials, further inquiries can be directed to the corresponding author/s.

## Author Contributions

JC took charge of the paper writing and data analysis. JL took charge of the technical guidance and supervision. SY took charge of literature research and providing research idea. ZD and XZ took charge of the microstructure characterization. WZ took charge of the room-temperature mechanical properties testing. JG and HW took charge of the preparation of samples. HS took charge of the hot rolling of alloy bars. ZY took charge of the texture evolution analysis.

## Conflict of Interest

The authors declare that the research was conducted in the absence of any commercial or financial relationships that could be construed as a potential conflict of interest.

## References

[B1] Banerjee D. Williams J. C. (2013). Perspectives on titanium science and technology. Acta Mater. 61, 844–879. 10.1016/j.actamat.2012.10.043

[B2] Cai S. Bailey D. M. Kay L. E. (2012). Effect of annealing and cold work on mechanical properties of β III titanium. J. Mater. Eng. Performance 21, 2559–2565. 10.1007/s11665-012-0302-4

[B3] Cai S. Daymond M. R. Ren Y. Bailey D. M. Kay L. E. (2013). Influence of short time anneal on recoverable strain of β III titanium alloy. Mater. Sci. Eng. A 562, 172–179. 10.1016/j.msea.2012.11.005

[B4] Chai L. Chen K. Zhi Y. Murty K. L. Chen L. Y. Yang Z. (2018). Nanotwins induced by pulsed laser and their hardening effect in a Zr alloy. J. Alloys Compounds 748, 163–170. 10.1016/j.jallcom.2018.03.126

[B5] Chai L. J. Wang S. Y. Wu H. Guo N. Pan H. C. Chen L. Y. (2017). α → β Transformation characteristics revealed by pulsed laser-induced non-equilibrium microstructures in duplex-phase Zr alloy. Sci. China Technol. Sci. 60, 1255–1262. 10.1007/s11431-016-9038-y

[B6] Chaves J. M. Florêncio O. Silva P. S. Marques P. W. B. Afonso C. R. M. (2015). Influence of phase transformations on dynamical elastic modulus and anelasticity of β Ti–Nb–Fe alloys for biomedical applications. J. Mech. Behav. Biomed. Mater. 46, 184–196. 10.1016/j.jmbbm.2015.02.030 25796065

[B7] Chen L. Shen P. Zhang L. Lu S. Chai L. Yang Z. (2018). Corrosion behavior of non-equilibrium Zr-Sn-Nb-Fe-Cu-O alloys in high-temperature 0.01 M LiOH aqueous solution and degradation of the surface oxide films. Corrosion Sci. 136, 221–230. 10.1016/j.corsci.2018.03.012

[B8] Chen L. Xu T. Wang H. Sang P. Lu S. Wang Z.-X. (2019). Phase interaction induced texture in a plasma sprayed-remelted NiCrBSi coating during solidification: an electron backscatter diffraction study. Surf. Coat. Technol. 358, 467–480. 10.1016/j.surfcoat.2018.11.019

[B9] Chen L.-Y. Cui Y.-W. Zhang L.-C. (2020). Recent development in β titanium alloys for biomedical applications. Metals 10:1139 10.3390/met10091139

[B10] Cheng J. Wang H. Li J. Gai J. Ru J. Du Z. (2020). The effect of cold swaging deformation on the microstructures and mechanical properties of a novel metastable β type Ti−10Mo−6Zr−4Sn−3Nb alloy for biomedical devices. Front. Mater. 7:228 10.3389/fmats.2020.00228

[B11] Cremasco A. Andrade P. N. Contieri R. J. Lopes E. S. N. Afonso C. R. M. Caram R. (2011). Correlations between aging heat treatment, ω phase precipitation and mechanical properties of a cast Ti–Nb alloy. Mater. Design 32, 2387–2390. 10.1016/j.matdes.2010.11.012

[B12] Cui C. Hu B. Zhao L. Liu S. (2011). Titanium alloy production technology, market prospects and industry development. Mater. Design 32, 1684–1691. 10.1016/j.matdes.2010.09.011

[B13] Cui W. F. Guo A. H. (2009). Microstructures and properties of biomedical TiNbZrFe β-titanium alloy under aging conditions. Mater. Sci. Eng. A 527, 258–262. 10.1016/j.msea.2009.08.057

[B14] Dong R. Li J. Kou H. Fan J. Zhao Y. Hou H. (2020). ω-Assisted refinement of α phase and its effect on the tensile properties of a near β titanium alloy. J. Mater. Sci. Technol. 44, 24–30. 10.1016/j.jmst.2019.10.031

[B15] Du Z. Guo H. Liu J. Cheng J. Zhao X. Wang X. (2020). Microstructure evolution during aging heat treatment and its effects on tensile properties and dynamic Young's modulus of a biomedical β titanium alloy. Mater. Sci. Eng. A 791:139677 10.1016/j.msea.2020.139677

[B16] Gao J. Knowles A. J. Guan D. Rainforth W. M. (2019). ω phase strengthened 1.2GPa metastable β titanium alloy with high ductility. Scripta Mater. 162, 77–81. 10.1016/j.scriptamat.2018.10.043

[B17] Guo H. Du Z. Wang X. Cheng J. Liu F. Cui X. (2019). Flowing and dynamic recrystallization behavior of new biomedical metastable β titanium alloy. Mater. Res. Express 6:0865d2. 10.1088/2053-1591/ab2421

[B18] Guo Y. Chen D. Cheng M. Lu W. Wang L. Zhang X. (2013). The bone tissue compatibility of a new Ti35Nb2Ta3Zr alloy with a low Young's modulus. Int. J. Mol. Med. 31, 689–697. 10.3892/ijmm.2013.1249 23338484

[B19] Ivasishin O. M. Markovsky P. E. Matviychuk Y. V. Semiatin S. L. Ward C. H. Fox S. (2008). A comparative study of the mechanical properties of high-strength β-titanium alloys. J. Alloys Compounds 457, 296–309. 10.1016/j.jallcom.2007.03.070

[B20] Kaur M. Singh K. (2019). Review on titanium and titanium based alloys as biomaterials for orthopaedic applications. Mater. Sci. Eng. C 102, 844–862. 10.1016/j.msec.2019.04.064 31147056

[B21] Laheurte P. Eberhardt A. Philippe M. Deblock L. (2006). Improvement of pseudoelasticity and ductility of ? III titanium alloy—application to orthodontic wires. Eur. J. Orthodontics 29, 8–13. 10.1093/ejo/cjl038 16954181

[B22] Lai M. J. Li T. Yan F. K. Li J. S. Raabe D. (2021). Revisiting ω phase embrittlement in metastable β titanium alloys: role of elemental partitioning. Scripta Mater. 193, 38–42. 10.1016/j.scriptamat.2020.10.031

[B23] Li H.-B. Chen M.-S. Tian Y.-Q. Chen L.-S. Chen L.-Q. (2020). Ultra-fine-grained ferrite prepared from dynamic reversal austenite during warm deformation. Acta Metallurgica Sinica 33, 290–298. 10.1007/s40195-019-00973-5

[B24] Li T. Kent D. Sha G. Cairney J. M. Dargusch M. S. (2016). The role of ω in the precipitation of α in near-β Ti alloys. Scripta Mater. 117, 92–95. 10.1016/j.scriptamat.2016.02.026

[B25] Li T. Kent D. Sha G. Dargusch M. S. Cairney J. M. (2015). The mechanism of ω-assisted α phase formation in near β-Ti alloys. Scripta Mater. 104, 75–78. 10.1016/j.scriptamat.2015.04.007

[B26] Li T. Kent D. Sha G. Liu H. Fries S. G. Ceguerra A. V. (2018). Nucleation driving force for ω-assisted formation of α and associated ω morphology in β-Ti alloys. Scripta Mater. 155, 149–154. 10.1016/j.scriptamat.2018.06.039

[B27] Nag S. Banerjee R. Srinivasan R. Hwang J. Y. Harper M. Fraser H. L. (2009). ω-Assisted nucleation and growth of α precipitates in the Ti−5Al−5Mo−5V−3Cr−0.5Fe β titanium alloy. Acta Mater. 57, 2136–2147. 10.1016/j.actamat.2009.01.007

[B28] Niinomi M. Nakai M. Hieda J. (2012). Development of new metallic alloys for biomedical applications. Acta Biomater. 8, 3888–3903. 10.1016/j.actbio.2012.06.037 22765961

[B29] Ohmori Y. Ogo T. Nakai K. Kobayashi S. (2001). Effects of ω-phase precipitation on β → α, α′′ transformations in a metastable β titanium alloy. Mater. Sci. Eng. A 312, 182–188. 10.1016/S0921-5093(00)01891-8

[B30] Qazi J. I. Marquardt B. Allard L. F. Rack H. J. (2005). Phase transformations in Ti−35Nb−7Zr−5Ta–(0.06–0.68)O alloys. Mater. Sci. Eng. C 25, 389–397. 10.1016/j.msec.2005.01.022

[B31] Rabadia C. D. Liu Y. J. Zhao C. H. Wang J. C. Jawed S. F. Wang L. Q. (2019). Improved trade-off between strength and plasticity in titanium based metastable β type Ti-Zr-Fe-Sn alloys. Mater. Sci. Eng. A 766:138340 10.1016/j.msea.2019.138340

[B32] Rack H. J. Kalish D. Fike K. D. (1970). Stability of as-quenched β-III titanium alloy. Mater. Sci. Eng. 6, 181–198. 10.1016/0025-5416(70)90048-0

[B33] Santhosh R. Geetha M. Saxena V. K. Nageswararao M. (2014). Studies on single and duplex aging of metastable β titanium alloy Ti−15V−3Cr−3Al−3Sn. J. Alloys Compounds 605, 222–229. 10.1016/j.jallcom.2014.03.183

[B34] Schmidt P. El-Chaikh A. Christ H. J. (2011). Effect of duplex aging on the initiation and propagation of fatigue cracks in the solute-rich metastable β titanium alloy Ti 38-644. Metall Mater. Trans. A 42, 2652–2667. 10.1007/s11661-011-0662-7

[B35] Shi R. Zheng Y. Banerjee R. Fraser H. L. Wang Y. (2019). ω-Assisted α nucleation in a metastable β titanium alloy. Scripta Mater. 171, 62–66. 10.1016/j.scriptamat.2019.06.020

[B36] Tang B. Chu Y. Zhang M. Meng C. Fan J. Kou H. (2020). The ω phase transformation during the low temperature aging and low rate heating process of metastable β titanium alloys. Mater. Chem. Phys. 239:122125 10.1016/j.matchemphys.2019.122125

[B37] Tang X. Ahmed T. Rack H. J. (2000). Phase transformations in Ti-Nb-Ta and Ti-Nb-Ta-Zr alloys. J. Mater. Sci. 35, 1805–1811. 10.1023/A:1004792922155

[B38] Vishnu J. Sankar M. Rack H. J. Rao N. Singh A. K. Manivasagam G. (2020). Effect of phase transformations during aging on tensile strength and ductility of metastable β titanium alloy Ti−35Nb−7Zr−5Ta-0.35O for orthopedic applications. Mater. Sci. Eng. A 779:139127 10.1016/j.msea.2020.139127

[B39] Wang L. Lu W. Qin J. Zhang F. Zhang D. (2009). Effect of precipitation phase on microstructure and superelasticity of cold-rolled β titanium alloy during heat treatment. Mater. Design 30, 3873–3878. 10.1016/j.matdes.2009.03.042

[B40] Wang P. Todai M. Nakano T. (2018). ω-phase transformation and lattice modulation in biomedical β-phase Ti-Nb-Al alloys. J. Alloys Compounds 766, 511–516. 10.1016/j.jallcom.2018.06.266

[B41] Weiss I. Semiatin S. L. (1998). Thermomechanical processing of β titanium alloys—an overview. Mater. Sci. Eng. A 243, 46–65. 10.1016/S0921-5093(97)00783-1

[B42] Xiang K. Chen L.-Y. Chai L. Guo N. Wang H. (2020). Microstructural characteristics and properties of CoCrFeNiNbx high-entropy alloy coatings on pure titanium substrate by pulsed laser cladding. Appl. Surf. Sci. 517:146214 10.1016/j.apsusc.2020.146214

[B43] Xiao J. F. Nie Z. H. Ma Z. W. Liu G. F. Hao F. Tan C. W. (2020). ω precipitation: deformation regulator in metastable titanium alloys. Mater. Sci. Eng. A 772:138687 10.1016/j.msea.2019.138687

[B44] Yang Z. N. Wang X. B. Liu F. Zhang F. C. Chai L. J. Qiu R. S. (2019). Effect of intercritical annealing temperature on microstructure and mechanical properties of duplex Zr-2.5Nb alloy. J. Alloys Compounds 776, 242–249. 10.1016/j.jallcom.2018.10.320

[B45] Zhang C. Ding Z. Xie L. Zhang L.-C. Wu L. Fu Y. (2017). Electrochemical and in vitro behavior of the nanosized composites of Ti-6Al-4V and TiO2 fabricated by friction stir process. Appl. Surf. Sci. 423, 331–339. 10.1016/j.apsusc.2017.06.141

[B46] Zhang L.-C. Chen L.-Y. (2019). A review on biomedical titanium alloys: recent progress and prospect. Adv. Eng. Mater. 21:1801215 10.1002/adem.201801215

[B47] Zhang L.-C. Chen L.-Y. Wang L. (2020). Surface modification of titanium and titanium alloys: technologies, developments and future interests. Adv. Eng. Mater. 22:1901258 10.1002/adem.201901258

[B48] Zheng Y. Choudhuri D. Alam T. Williams R. E. A. Banerjee R. Fraser H. L. (2016a). The role of cuboidal ω precipitates on α precipitation in a Ti-20V alloy. Scripta Mater. 123, 81–85. 10.1016/j.scriptamat.2016.06.004

[B49] Zheng Y. Williams R. E. A. Wang D. Shi R. Nag S. Kami P. (2016b). Role of ω phase in the formation of extremely refined intragranular α precipitates in metastable β-titanium alloys. Acta Mater. 103, 850–858. 10.1016/j.actamat.2015.11.020

[B50] Zhu C. Lv Y. Qian C. Qian H. Jiao T. Wang L.. (2016). Proliferation and osteogenic differentiation of rat BMSCs on a novel Ti/SiC metal matrix nanocomposite modified by friction stir processing. Sci. Rep. 6:38875. 10.1038/srep38875 27958394 PMC5153627

